# Consensus on a conversation aid for shared decision making with people with intellectual disabilities in the palliative phase

**DOI:** 10.1111/jar.12898

**Published:** 2021-06-01

**Authors:** Hanna W. Noorlandt, Ida J. Korfage, Irene Tuffrey‐Wijne, Dederieke Festen, Cis Vrijmoeth, Agnes van der Heide, Michael Echteld

**Affiliations:** ^1^ Department of Public Health Erasmus Medical Center Rotterdam Rotterdam, Zuid‐Holland the Netherlands; ^2^ Faculty of Health, Social Care and Education Cranmer Terrace London Kingston University & St. George’s University of London London UK; ^3^ Department of General Practice, Intellectual Disability Medicine Erasmus Medical Center Rotterdam Rotterdam, Zuid‐Holland The Netherlands; ^4^ Eleos/De Hoop GGZ Centre for Research and Innovation in Christian Mental Health Care Amersfoort, Utrecht The Netherlands; ^5^ Expertise Centre Caring Society Avans University of Applied Science Breda, Noord‐Brabant The Netherlands; ^6^ Prisma Foundation Waalwijk, Noord Brabant Netherlands

**Keywords:** care for people with intellectual disabilities, conversation aid, Delphi study, end‐of‐life, palliative care, shared decision‐making

## Abstract

**Background:**

Little is known about how to involve people with intellectual disabilities in making decisions about treatment and care in their palliative phase. We aimed to reach a consensus about a shared decision‐making (SDM) conversation aid for people with intellectual disabilities, relatives, and healthcare professionals.

**Methods:**

In a Delphi process, an expert panel of 11 people with intellectual disabilities, 14 relatives, and 65 healthcare professionals completed online questionnaires about the relevance and feasibility of a draft conversation aid.

**Results:**

In Round 1, components were rated as (very) relevant by 70–98% of participants (M = 87%). In Round 2, after amending the aid in response to feedback, relevance ratings were 67–97% (M = 90%) and feasibility ratings 66–86% (M = 77%). The final version consists of four themes: who are you; illness/end‐of‐life; making decisions; and evaluating the decision.

**Conclusion:**

The consensus‐based conversation aid is considered sufficiently relevant and feasible to be implemented in practice.

## 1 INTRODUCTION

Shared Decision Making (SDM) is currently seen as the preferred practice when medical decisions are needed. Ideally, doctors, care providers, and patients discuss possible care and treatment options. Patients’ preferences and values are weighed and the best possible choice is jointly made by the patient and health care professional (Stiggelbout et al., 2012). Decision‐making processes can become more difficult if a person with an intellectual disability cannot actively participate in these conversations. Watson et al developed a supported decision‐making framework especially for these situations, focussing on how to involve people with severe and profound intellectual disabilities meaningfully (Watson et al., 2017). They explain how important it is to have a good, emotionally involved support network, in which the key members are well aware of the preferences and wishes of the person with an intellectual disability. This applies not only to people with severe and profound intellectual disabilities, but to anyone who has to make major care or treatment decisions, including palliative care and end‐of‐life decisions. Palliative care can, through the process of SDM, be better aligned to the values and needs of a person. The World Health Organization defines palliative care as “*an approach that improves quality of life of patients and their families facing the problem associated with life*‐*threatening illness”(*WHO, 2020 *)*. When the need for palliative care is recognised in good time, it allows people to discuss their preferences and adjust their care to these preferences.

During the last few decades, palliative care has become recognised as a central tenet to improving end‐of‐life care for people of all ages. There has been less focus and less research into palliative care for people with intellectual disabilities. In 2016 the European Association for Palliative Care published consensus‐based guidelines on how policy, research and practice can improve palliative care for people with intellectual disabilities (Tuffrey‐Wijne et al., 2016). One of these norms states that people with intellectual disabilities should be involved in end‐of‐life decision‐making and should have all the support they need to do so (Tuffrey‐Wijne et al., 2016). People with intellectual disabilities have the right to be involved in decision‐making processes about their care and treatment (United Nations, 2006). Article 12 of the Convention on the Rights of Persons with Disabilities (United Nations, 2006) acknowledges the right people with intellectual disabilities have “to be recognised by law as a person equal to others” (United Nations, 2006). Nevertheless, studies have found that people with intellectual disabilities are often not included in decision‐making processes (Kirkendall et al., 2017; Voss et al., 2019; Wagemans et al., 2013).

Timely identification of the palliative care phase is necessary in order to ensure that palliative care is properly aligned to the values and wishes of a person with intellectual disability (Vrijmoeth, Christians, Festen, Groot, Tonino, et al., 2016; Vrijmoeth, Christians, Festen, Groot, van der Heide, et al., 2016). The timely identification of the palliative phase is necessary to enable the beginning of shared decision‐making conversations about care and treatment in the palliative phase. By beginning these conversations as early as relevant and possible, there is more time to find out the wishes and preferences of the person with an intellectual disability and to better align the care practices to these preferences. Regularly revisiting these conversations is important as wishes and preferences can change significantly in the palliative phase (Kirkendall et al., 2017). Identification of the palliative phase is in people with intellectual disabilities is complicated due to a range of factors, such as the difficulty of identifying pain and other symptoms (Tuffrey‐Wijne et al., 2016), the high prevalence of multimorbidity and the difficulties in reciprocal communication (Vrijmoeth et al., 2018).

People with intellectual disabilities are often not actively involved in decision‐making processes in the last phase of their lives due to communication challenges, assumptions that people with intellectual disabilities will not be able to handle difficult conversations, assumed lack of capacity and the lack of advanced care planning skills of healthcare professionals (Kirkendall et al., 2017; Voss et al., 2019; Wagemans et al., 2010). Only two out of ten papers in a scoping review showed the involvement of people with an intellectual disability in the end‐of‐life decision‐making process, and in those two papers, the nature of involvement was unclear (Noorlandt et al., 2020). However, there are clear indications that people with intellectual disabilities want to be involved in the decision‐making process around their care (Tuffrey‐Wijne et al., 2016). Watson's supported decision‐making framework showed that this was possible even for people with a severe and profound intellectual disability (Watson et al., 2017). In recent years there has been a growing body of literature around what supported decision‐making can mean for people with intellectual disabilities, including the challenges and possibilities (Craigie, 2015; Devi, 2013; Kohn & Blumenthal, 2014; Kripke, 2016; Lotan & Ells, 2010; Scholten & Gather, 2018).

McKenzie et al. found that involving people with intellectual disabilities in Advance Care Planning (ACP) had positive outcomes in terms of discussing matters at people's own pace, getting support to make their own choices, adapting the process to who they are, and, most importantly, to continue to shape their life the way they want to (McKenzie et al., 2017). However, significant challenges to involving people with intellectual disabilities in ACP have been identified. Healthcare professionals have indicated that they find it difficult to start conversations concerning illness and death (Voss et al., 2017). It can be difficult to identify someone's preferences when there are communication challenges or when people can only express themselves non‐verbally (Kirkendall et al., 2017; Voss et al., 2019; Vrijmoeth, Barten, et al., 2016). Hesitations of family members and healthcare professionals to speak about serious illness and impending death can lead to reluctance to involve people with intellectual disabilities in conversations about their end‐of‐life care. Starting such conversations may be difficult for people with intellectual disabilities themselves as well, as they may not always understand all implications of their disease (Tuffrey‐Wijne, 2013). Still, it is imperative to try to take preferences for treatment and care of people with intellectual disabilities into account. SDM can be a useful approach, because it offers the space to look at all the options available from all possible perspectives. In this way, it is possible to make choices based on the values and wishes of the person with an intellectual disability. Using a SDM tool in this process can be valuable, as it has been found that using instruments or aids can help improve the care for people with intellectual disabilities (Bekkema et al., 2015; McKenzie et al., 2017; Vrijmoeth et al., 2018). An example of this is PALLI, an instrument to facilitate the marking of the palliative phase in people with intellectual disabilities (Vrijmoeth et al., 2018).

We believe, therefore, that an aid to support SDM with people with intellectual disabilities in the palliative phase would be of significant benefit to the quality of life of these individuals, as well as their families. In this paper, we describe the development of the content of an aid for SDM in people with intellectual disabilities in the palliative phase. More specifically, we aimed at a consensus regarding the relevance and feasibility of a draft conversation aid.

## 2 METHODS

### 2.1 Development design

After comprehensive literature searches and network consultation (Noorlandt et al., 2020), it appeared that there were no suitable SDM tools available. Models that were developed for people with intellectual disabilities did not focus on the palliative care phase (Douglas & Bigby, 2020; Sullivan et al., 2018). We, therefore, looked for an existing SDM model as a starting point, developed for another target group where communication difficulties were also an issue. We selected the SDM model by Van de Pol for frail older people with high levels of multimorbidity (van de Pol et al., 2016), for a number of reasons. Its development was based on a comprehensive 3‐round Delphi consensus procedure with 75 patients and professionals. They reached 91% and 76% agreement with respect to importance and feasibility of their SDM model. The Van de Pol model is therefore considered promising by other researchers, it is currently being adapted for use with different target populations, namely older patients with multiple chronic conditions and people with dementia (Pel‐Littel et al., 2019, 2020; Tilburgs et al., 2020). The model explicitly takes the involvement of informal caregivers into account, which is also essential among people with intellectual disabilities. The model focuses on the personal context of the patient and a continuous dialogue between the patient, possibly an informal caregiver and health care professional in decision‐making processes about treatment and care. Overall, Van de Pol's model seemed a promising fit with our target population. See Box 1 for the six steps of the Van de Pol model. In brief, these steps are: (1) Preparation; (2) Goal talk; (3) Choice talk; (4) Option Talk; (5) Decision talk; (6) Evaluation. This approach allows room for the values and care goals of patients and it supports health care professionals in applying a more patient‐centered approach. To adapt this model to a conversation aid for people with intellectual disabilities, we conducted a scoping review about the involvement of people with intellectual disabilities in decision‐making processes in the last phase of life as a guideline (Noorlandt et al., 2020), using search terms related to people with intellectual disabilities, shared decision‐making, end‐of‐life‐decision‐making, and palliative phase. From this literature, we collated themes that we considered important for our conversation aid. We discussed these themes with various experts. We adapted the model based on this literature research, the conversations with experts and a two‐step consensus procedure.

BOX 1 **The six steps of Van de Pols SDM model (van de Pol et al., 2016).**

**Table:** 

1. Preparation:
History: has the patient already documented anything with regard to advance care planning, treatment e.g. Problem analysis: what are the current problems of the patient.
2. Goal talk:
Is the patient capable of making choices? Does the patient want to make these choices? If not, what has designated to makes these choices for the patient? What are important values and care goals for the patient?
3. Choice talk:
Summarise the earlier described steps. Explain that there are several treatment options and offer choice. Encourage the patient to express their treatment aims.
4. Option talk:
The chosen treatment options will be discussed.
5. Decision talk:
Focus on the patients preferences. Connect to the values, care and treatment goals that are important to the patient. Make a decision.
6. Evaluation
Evaluate the SDM process. If everybody is satisfied a treatment plan can be prepared.

### 2.2 Consensus procedures

#### 2.2.1 Consultation

To ensure that the content of the conversation aid was relevant and clear to people with intellectual disabilities, we discussed the first draft during a focus group of five people with intellectual disabilities. They were all members of the client panel of a care facility. Members of a client panel represent the common interests of clients living in or connected to that care facility. After we processed their feedback, we asked three relatives of people with intellectual disabilities to pilot our conversation aid. We then consulted people with intellectual disabilities on the applicability of the content using focus group interviews.

### 2.3 Delphi procedure

We designed a Delphi procedure using the COMET handbook (Williamson et al., 2017), intended to systematically reach consensus on the relevance and feasibility of each component in the draft conversation aid. As is usual in Delphi procedures, we asked in several consecutive rounds a panel of experts to give their opinion on given topics. After each round, we communicated the outcome of the questionnaire to all the experts, who were invited to reconsider their ratings (Williamson et al., 2017). We repeated these steps until we reached sufficient consensus, which was defined as 70% agreement.

After each Delphi round, we presented the results of our analysis to our advisory group. This advisory group is involved in the larger study of which this Delphi study is a part and has an advisory role in the decisions that must be made within the project. The group has expertise in the areas of palliative care, people with intellectual disabilities and SDM/Advance Care Planning (ACP). This advisory group oversees the consensus procedures of our Delphi procedure. One member of this advisory group, Irene Tuffrey‐Wijne also has her own Group for Research Advice, Sharing and Support, the GRASSroots group, consisting of people with intellectual disabilities who meet monthly to talk about death and dying. Irene asked five members of the GRASSroots group for feedback on the conversation aid, which was recorded and incorporated in the subsequent development. Adjustments were made based on the obtained comments.

### 2.4 Selection

We invited experts in the following fields to participate in the Delphi panel: relatives of people with intellectual disabilities; shared decision‐making and advance care planning (ACP) experts; researchers with expertise in the field of people with intellectual disabilities, palliative care and/or ACP; and palliative care experts. We invited professionals involved in the daily care of people with intellectual disabilities. We also invited experiential experts with intellectual disabilities to join our Delphi panel, which means “(…) someone who, based on personal and collective experiences, is able to broaden knowledge from experience in any form, and pass it on to others” (van der Eerden, 2017). Due to international differences in healthcare systems for people with intellectual disabilities and cultural differences in talking about death, we decided to use a panel focussed on the Netherlands. The researchers used their own networks to invite members to the Delphi expert panel. We specifically searched for relatives of people with intellectual disabilities, experts in the field of SDM/ACP and palliative care experts.

### 2.5 Design and analysis

We presented our draft conversation aid to the experts in our Delphi panel. We asked them to quantify the relevance of the different components in the conversation aid, on a fully labelled 5‐point Likert‐scale in which 1 indicated “not relevant at all” and 5 indicated “very relevant”. We opted for this scale because these are relatively easy to manage for people who are not familiar with completing structured questionnaires (Bayer & Wittink, 2003; Revilla et al., 2014; Weijters et al., 2010). To be retained for the next round, a single component had to be rated as 4 or 5 (‘relevant’ or ‘very relevant’) by at least 70% of respondents and as 1 or 2 (‘not relevant at all’ or ‘not relevant’) by at most 15% of the participants. If more than 70% of the participants rated a component as 1 or 2 and less than 15% of the respondents rated it as 4 or 5, the component would be removed from the conversation aid (Williamson et al., 2017). We decided that if there were fewer than four components that had not yet been agreed on, we would discuss these further in our advisory group. It was possible to provide feedback considering every component of the conversation aid. New Delphi rounds will be initiated until consensus is reached. When the scores in a new round differ less than 3% from the previous round, no new round is needed (Williamson et al., 2017).

### 2.6 Procedure

We invited all experts individually to participate in de Delphi questionnaire by e‐mail, explaining our study, the Delphi procedure and the voluntary nature of this study. We explained that the data from the questionnaire were treated confidentially and processed anonymously, and that we considered participation in this study as informed consent. In March 2019, we presented the draft conversation aid to the experts who agreed to participate in the Delphi study. We used an online questionnaire in a secure online environment in which the questionnaires could be stored. First, the aim of the study, the target group and the characteristics of the conversation aid were described. In the second part, the concept aid was introduced, consisting of 18 components. Experts were asked to rank each component for relevance.

Subsequently, panel members were asked what they thought of the conversation aid in general, whether the overall aim was clear, and whether any components were still missing or were considered unnecessary. They were also asked what was needed to implement this conversation aid in practice. After 10 days, participants received a reminder—up to two reminders per round. The hyperlink for the next Delphi round was only sent to panel members who completed the first Delphi round.

We received approval from the Erasmus University Medical Center research ethics committee (METC‐2018–1683) to perform this Delphi study. Every time the Delphi questionnaire was sent to the online panel, we also met with a group of experiential experts with intellectual disabilities to complete the same questionnaire on paper. Where necessary, the questions were explained and the answers were combined in the analysis with the answers from the other online experts.

## 3 RESULTS

### 3.1 First draft conversation aid

We adapted the Van de Pol model based on discussions among the authors, conversations with experts and other conversation aids found in the literature. The first draft version of the conversation aid consisted of 18 components (see Table 1), each component having an accompanying black and white icon.

**TABLE 1:** Draft aid for SDM with people with intellectual disabilities in the palliative phase

Steps	Potentially helpful phrases
Step 1.	You and I agree: it's about you. So you can say everything you think and feel. Nothing is strange or wrong.
Step 2.	Together we make a group of people. This group includes people who are very important to you and who you trust. We call this group of people the core team.
	Together with you, we ask these people to help you make the difficult choice.
Step 3.	How is it for you to be ill? Do you know what is going on with you?
	How do you feel about that? You can tell me. And what do we
	need to know to help you? Do you know enough about your
	situation? What do you want to know to make a good choice? You can tell me. You and I talk together and listen to each other.
Step 4.	Do we have all the information we need to make a good decision?
	Then we talk together: do you want to hear all information at once?
	Or do you want to get a little bit of information at a time? It is up to you.
Step 5.	We choose a key person together with you. That is someone
	with whom you can talk very well and who can explain things well to you.
	He or she becomes very important to you and us when we make choices.
Step 6.	What is the best way to explain things to you? Are pictures good for you?
	Or do you like it when someone uses words to explain things to you?
	You and I choose the best way to explain things to you.
Step 7.	It can be difficult to think about getting information. Your core team will help you with this.
Step 8.	Where shall we talk about the decision we are going to make together?
	Where do you feel safe and secure? Maybe in the living room, or in your own room.
	Or at your parent's house. You decide.
Step 9.	What things make you happy? What do you enjoy? What do you like to do?
	We talk about this together.
Step 10.	Have you ever had to make a difficult choice before?
	Who helped you make the choice then?
	What did you do yourself to make the choice?
	You and I talk together and listen to each other.
Step 11.	What do you think of the decision we are going to make together?
Step 12.	Do you ever think about dying? What do you feel when you think about death?
	Do you have questions that you want to talk about?
	If you were going to die, what would you wish for?
Step 13.	We talk together to see if we have told you everything in the right way. Then we will help you remember all the information.
Step 14.	Do you want to be there when your core team talks about your decision?
	Or do you want someone else to tell them about the things that matter to you?
Step 15.	Together we look which decision is best for you.
Step 16.	We are now making the decision together.
Step 17.	Who needs to know about your decision? Then they can better understand and help you.
	What does staff need to know about you, so that they can support you well?
Step 18.	We talk again about your decision. What was it like for you? What do you think of the help you got with this?
	What do you think about your situation, now that you have made your choice?

This draft version was discussed during a focus group of people with intellectual disabilities. The focus group indicated, for example, that the accompanying icons with the conversation aid are of great importance, because not every person with an intellectual disability is equally capable of reading and interpreting a text. They said that the images should show that healthcare professionals will support you in making decisions in this decision‐making process. However, they indicated that the black and white icons were too abstract. Based on this feedback we adjusted the icon. Thereafter, three relatives’ pilot tested the conversation aid. They indicated that trust between healthcare professionals and people with intellectual disabilities is most important when working with the conversation aid. In addition, they indicated that there should be a stronger emphasis on the fact that this aid revolves around the person with intellectual disability and that questions about end‐of‐life wishes and funeral wishes should be separated.

### 3.2 Delphi procedure

We invited 98 experts to join the Delphi panel, of whom 90 (91%) completed the first questionnaire. The Delphi panel consisted of 11 experiential experts with intellectual disabilities, 14 relatives of people with intellectual disabilities, 2 shared decision‐making and advance care planning experts, 10 researchers, 22 healthcare professionals, 10 palliative care experts, 6 managers, 5 physicians, 2 policy advisers, 2 nurses, 1 mental caretaker, 1 teacher, 1 physiotherapist, 1 behavioural expert, 1 volunteer and someone with a different background. All participants came from the Netherlands.

### 3.3 Delphi Round 1

The components were considered “relevant” or “highly relevant” by 70% to 98% of panel members (Mean =87%). The first author (HN) selected recurring themes that emerged in the open text comments. The advisory group discussed the feedback and, where necessary, asked the relevant experts for further explanations. We used the feedback to adjust the conversation aid. Based on the feedback, we combined eight components into four components. We adjusted a number of terms, for example, we replaced ‘core team with ‘Circle of Support, ‘decision’ with ‘choice’ and ‘parents’ with ‘family’. Furthermore, additional explanations were included about the application of the conversation aid. In addition, the order of the components was also adjusted based on the obtained comments and feedback. It became clear that more clarity was needed about the premises of the conversation aid. Furthermore, it became apparent that there was a need to make the conversation aid flexible to enable use for people with all levels of intellectual disabilities.

### 3.4 Delphi Round 2

Seventy‐three people participated in the second Delphi round (a response rate of 81%). Components were considered “relevant” or “highly relevant” by 67% to 97% of panel members (Mean =90%). We removed the component “making choices” that was considered relevant by 67% of the panel. In the second Delphi round, we also asked panel members to assess the relevance of each component of the conversation aid. Components were rated as “feasible” or “very feasible” by 66% to 86% of the panel (Mean =77%). In the open text comments, experts indicated that the flexibility of the conversation aid lies in the possibility to adapt the aid to the individual, their situation and preferences. In addition, it became clear that the conversation aid should be used in a dynamic, non‐linear way. In other words, people can decide, in consultation with each other, what components of the conversation aid should be discussed and in what order. Experts indicated that people's wishes and preferences can change significantly in the palliative phase. This aid should therefore be used as part of a repetitive process, in which wishes and preferences are reviewed regularly. It also became clear that the conversation skills of the healthcare professionals are extremely important when using the conversation aid. This could include being open to all possible answers, listening carefully, asking questions and indicating when you do not understand something.

**FIGURE 1:**
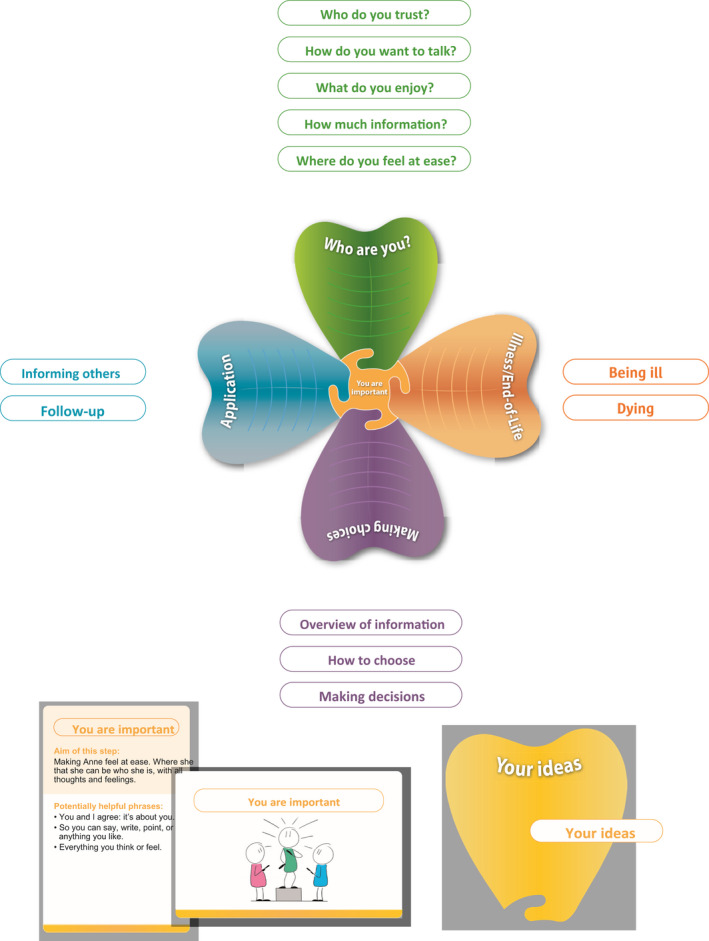
Aid for SDM with people with intellectual disabilities in the palliative phase

The authors presented the results of this analysis to the advisory group. Irene Tuffrey‐Wijne presented the feedback from her GRASSroots group about the conversation aid. The GRASSroots group's findings were consistent with the open text comments of the Delphi panel. The project group discussed this feedback. It was decided to order the different components in four different themes: who are you; illness/end‐of‐life; making choices; and application. We also introduced a generally applicable principle: ‘you are important’. See Box 2 for descriptions of the different themes. Based on the responses from the Delphi panel, the GRASSroots and advisory group we decided to develop conversation cards for each of the 13 components of the aid. The 13 conversation cards were provided with an illustration on the front and phrases on the back that can help healthcare professionals to start the conversation. See Table 2 for the potentially helpful phrases. We referred to the person with intellectual disability by using the name ‘Anne’, rather than using the words “person with intellectual disability.” See Figure 1 for the aid for SDM with people with intellectual disabilities in the palliative phase.

**TABLE 2:** Aid for SDM with people with intellectual disabilities in the palliative phase

Components	Potentially helpful phrases:
You are important	*You and I agree: it's about you. *So you can say, write, point or draw anything you like. *Everything you think and feel.
Who do you trust	*It can be good to talk to people you trust. *Who is important to you?
	Who else is important to you? Then you and me choose a group of people together.
	*This group is called a Circle of Support. *This group contains your key person and other people who are very important to you and whom you trust.
How do you want to talk	*What is the best way to explain things to you? *Are pictures good for you? Or do you like videos that explain things?*Or do you like it when someone uses words to explain things to you? *You can choose more than one way. *What is the best way for you to explain something to others? * You and I choose the best way(s) to explain things to you.
What do you enjoy?	*What things make you happy? *What do you enjoy? *What do you like to do? *What is important to you? *We talk about this together.
Hou much information do you want to receive?	*You need information to make a good choice. *You need to know what the doctor thinks about your current health status and what choices you can make, for example are there treatments you can get. *Do you want to know this, or not? *Do you want to hear all the information at once or do you want to get a little bit of information at a time? It is up to you.*It can be difficult to think about getting information. Your Circle of Support will help you with this.
Where do you feel at ease?	*Where shall we talk about the choice(s)? *Where do you feel safe and secure? *Maybe in the living room, or in your own room. Or at home with family. You decide. *You can always aks your questions, wherever and whenever you want.
Being ill	*What is the matter with you? *How do you feel? You can tell me. *What do you find the hardest about being ill? *Are there any nice things about being ill? Which things do you like? *Do you want to know more about your illness and what it means for you? *You can tell me. You and I talk together and listen to each other.
Dying	*Do you ever think about dying? What are you thinking then? *What do you feel when you think about death? *Do you have questions that you want to talk about? *If you were going to die, what would you wish for?
Overview of information	*We talk together to see if we have told you everything in the right way. *Then we will help you remember all the information. *If you have understood all information correctly, we will make a choice together. How do you feel about that? *You do not have to make that choice on your own, your Circle of Support can help you with this.*Do you need anything else to make a choice?
How do you want to make choices	*Are you ready to make a choice? *We will make the choice(s) together with you. *Do you want to be there when your Circle of Support talks about your choice? Or do you want someone else to tell them about the things that matter to you?
Making decisions	*We make the decision(s) together and we help you.
Telling others about your choice	*Who needs to know about your choice? Who else would you like to know about it? *Together, we will tell those people about your choice(s). *Then they can better understand and help you. *What does staff need to know about you, so that they can support you well?
Follow‐up	*We talk again about your choice. *What was it like for you? *What do you think about your choice? *What do you think of the help you got with this? *What do you think about your situation, now that you've made your choice?

BOX 2 **Descriptions four themes in the aid for SDM with people with intellectual disabilities in the palliative phase**

**Table:** 

Four themes	
Who are you?	This theme collects the information that is needed to make a good choice together. The theme examines who Anne trusts, how she wants to talk, what she enjoys, how much information she wants to get and where she feels safe and comfortable.
Illness/ End‐of‐life	In this theme information is collected to find out what Anne knows about her illness and what she wants to find out about it. And what her questions and wishes are about death and dying.
Making choices	In this theme the information is collected that is needed to find out if Anne has understood all the information correctly and if Anne is ready to make a choice. If so, the decision is actually made.
Application	This theme examines who must be informed of the decision that has been made. And, if applicable, consider whether the decision made is still the correct decision or needs to be revised

## 4 DISCUSSION

The aim of this study was to develop a consensus‐based tool for shared decision‐making (SDM) with people with intellectual disabilities in the palliative phase. We conducted a two‐round Delphi consensus procedure. The components of the tool we developed were considered “relevant” or “highly relevant”. The feedback from the expert panel led to improvements, for instance with respect to terminology.

The final conversation aid consists of four themes (“Who are you”, “Illness/End‐of‐life”, “Making choices” and “Application”) and has a guiding premise. Every conversation starts with the premise “you are important”. In this way, we try to create a safe environment where people are able to say what really matters to them. Each theme has different components that do not have to be followed in any particular order. To clarify this, the different components have names rather than numbers. In the theme “Who are you” information is collected to find out who the person with intellectual disability trusts, how they want to talk, what they enjoy, how much information they want and where they feel safe. In the theme “Illness/End‐of‐life”, information is collected to find out what the person with an intellectual disability knows and wants to know about their illness. The theme “Making choices” investigates if the person with an intellectual disability understood all the information and if they are ready to make a choice. The theme “Application” examines who needs to be informed about the decision that has been made, and investigates whether the decision is still right or needs to be revised.

The conversation aid should be used in a flexible way and could be applicable for people with all levels of intellectual disabilities. It is possible to adapt the aid to the person's preferences and situation. In addition, the conversation aid is not intended to be used in a single conversation, but in an ongoing decision‐making process, providing room for changes in points of view and situations. Good conversation skills are important for healthcare providers in these conversations. These could include being open to all possible answers, listening very carefully and asking questions.

This is the first conversation aid that was developed specially for people with intellectual disabilities in the palliative phase. The personal context of the patient will be taken into account in a continuous dialogue between the patient and health care professional in the decision‐making process. By using the four different themes in our conversation aid it is possible to assess the wishes and preferences of people with intellectual disabilities. In this way, we echo Van de Pol's patient‐centered approach based on values and goals of patients, but we have developed it further. Communication difficulties in people with intellectual disabilities are common, but Van de Pol's model was not designed for extracting values and preferences of people with intellectual disabilities. Moreover, Van de Pol's model focused mainly on medical decisions and was not developed for the palliative phase. Whilst our aid could be applied to all possible issues in the palliative phase that require a decision.

For people with intellectual disabilities, just as for anybody else, it is important to involve the inner circle of the person in decision‐making processes. We recommend healthcare professionals to use the conversation aid with the person with intellectual disabilities and their parents and other important people that are close to the person with an intellectual disability. Together they can ensure that the decision made is aligned with the preferences and values of the person with an intellectual disability. Looking at small gestures, facial expressions, earlier medical experiences and life history of a person with intellectual disabilities, can show the wishes and preferences someone has (Bekkema et al., 2015; Watson et al., 2017).

### 4.1 Strengths and limitations

This study has several strengths. Firstly, we used the COMET handbook which ensured complete and transparent reporting of our Delphi procedure and proper following of the Delphi procedure (Williamson et al., 2017). Secondly, the members of the Delphi expert panel represented a broad spectrum of perspectives. Thirdly, the involvement of experiential experts with intellectual disabilities ensured that the target group was involved in developing the conversation aid from the very start. Furthermore, we work together with an experiential expert as a co‐researcher with intellectual disabilities throughout the entire project. Fourthly, we had a multidisciplinary and specialist advisory group that advised us throughout the Delphi process. Fifthly, we used van de Pol's SDM model for frail older patients with multiple morbidities as a basis for our conversation aid (van de Pol et al., 2016).

This study also has several limitations. Firstly, 81% of the experts who took part in the first Delphi round also participated in the second Delphi round. The dropout rate of participants in the second Delphi round may affect the results. However, the results of the first round did not indicate differences in answers between the two groups. Secondly, the Delphi panel consisted of Dutch experts, which may require some degree of cultural adaptation of the tool for the care for people with intellectual disabilities in other countries than The Netherlands. Thirdly, 11 experiential experts with intellectual disabilities were involved in the Delphi panel. The tool is developed for healthcare professionals. An over‐representation of professionals is therefore understandable in this case. Fourthly, the current conversation aid may need adjustments to make it suitable for people with intellectual disabilities who have an auditory and/or visual impairment. Finally, to enable participation in the study people with intellectual disabilities had to have reasonable verbal ability, both receptive and expressive, thereby excluding people with more severe communication difficulties.

### 4.2 Practice implications

With this study, we provide professionals working with people with intellectual disabilities with an aid to initiate SDM; an essential part of palliative care. To use this aid effectively, appropriate training in using the aid is required. Furthermore, we recommend applying this aid at an early stage of the palliative phase. Timely recognition of the palliative phase is necessary to enable this. It is therefore recommended to adopt palliative care policies in health care for people with intellectual disabilities. This could be done by providing training in palliative care, supporting timely recognition of the palliative phase by using special screening tools such as PALLI or the Surprise Question (Vrijmoeth et al., 2018), and by promoting the importance of palliative care in care policy for people with intellectual disabilities. Readers who are interested in using our conversation aid can contact us by email at Samenspraakstappenplan@erasmusmc.nl.

## 5 CONCLUSION

We reached a consensus on an SDM aid that is suitable for healthcare professionals, families, and people with intellectual disabilities. The aid consists of different components, which can be used in a dynamic way, suitable for people with all levels of intellectual disabilities in the palliative phase. The conversation aid is developed by relatives, people with intellectual disabilities, healthcare professionals and researchers, which means there are favourable preconditions for the implementation of this aid in practice. In addition, our aid could be applied to all possible issues in the palliative phase that require a decision. Panel members consider the conversation aid relevant and feasible for use in practice. Testing the conversation aid in practice is an important next step.

## CONFLICT OF INTEREST

The manuscript, or part of it, neither has been published nor is currently under consideration for publication by any other journal. All authors (Hanna Noorlandt, Michael Echteld, Irene Tuffrey‐Wijne Dederieke Festen, Cis Vrijmoeth, Agnes van der Heide and Ida Korfage) have read the manuscript and approved its submission to the Journal of Applied Research in Intellectual Disabilities. They all declare that they have no competing interests.

## AUTHOR CONTRIBUTIONS

HN, IK and ME all conceived and designed the study, HN, IK and ME designed the questionnaires and analysed the data. All authors revised and approved the final version of this paper.
